# Editorial: Functional devices and biosensors

**DOI:** 10.3389/fbioe.2025.1721681

**Published:** 2025-11-07

**Authors:** Zetao Chen, Qian Chen

**Affiliations:** School of Disaster and Emergency Medicine, Tianjin University, Tianjin, China

**Keywords:** biosensors, functional devices, advanced materials, artificial intelligence, biomedical engineering (BME)

The rapid development of functional devices and biosensors is reshaping biomedical research and medical practice, which was drove by the innovations of materials science, imaging technology, and artificial intelligence (AI), (Wu et al., 2023). The intersection of these disciplines has yielded devices that not only sense biological signals but actively modulate physiological processes, while biosensors have become increasingly precise, adaptable, and clinically relevant (Lin et al., 2021). The collected articles highlighted in this Research Topic exemplified the boundaries of what these technologies can achieve, each contributing unique insights into how functional devices and biosensors can address critical challenges in healthcare, from cellular regulation to high-resolution imaging, intelligent data analysis, and real-time patient monitoring. Together, they underscore a shared vision: to create technologies that are not only technically sophisticated but biologically compatible, clinically actionable, and seamlessly integrated into routine care.

Materials innovation forms the foundation of next-generation functional devices. Franceschelli et al. demonstrate the potential of material-driven bioeffects in their study of graphene quantum dot (QD) devices (Franceschelli et al.). Their wearable, battery-free device emits electromagnetic fields, demonstrating its ability to alleviate oxidative stress in hydrogen peroxide-activated Jurkat T cells by modulating antioxidant enzymes like superoxide dismutase (SOD) and catalase (CAT). This work underscores how engineered nanomaterials can serve as bioactive functional units actively manipulate cellular pathways, bridging physical stimuli (electromagnetic fields) and biological responses (redox balance). Such materials not only expand the toolkit for non-pharmacological interventions but also inspire the design of biosensors that leverage quantum effects for sensitive biological signal transduction. These devices transcend passive monitoring, offering non-pharmacological interventions for conditions like chronic inflammation. However, scalability and long-term biocompatibility warrant deeper exploration, especially for *in vivo* translation.

Advances in computational methods are equally transformative. High-resolution imaging and detection are critical for biosensors to capture fine-grained biological information. Lin et al.’s review details how deep learning (DL) overcomes inherent limitations in light-field microscopy (LFM), enabling high-resolution 3D reconstruction of dynamic biological processes (Lin et al.). Traditional LFM suffers from low efficiency and artifacts, but DL architectures like LFMNet and F-VCD achieve near-confocal resolution (0.086 μm) and real-time processing (50 ms/frame). Light field microscopy, empowered by deep learning, provides a powerful platform for biosensors requiring spatiotemporal resolution, for example, tracking dynamic cellular processes or mapping tissue microstructures. This integration of imaging and AI not only improves detection accuracy but also enables real-time analysis, a key demand for point-of-care biosensors. Intelligent data processing is increasingly central to extracting meaningful characteristic parameters from biosensor outputs. Pan et al. proposed MIPC-Net, a deep learning model with a mutual inclusion mechanism for precise boundary segmentation in medical images, achieving superior performance in organ and lesion segmentation (e.g., 2.23 mm reduction in Hausdorff Distance on the Synapse dataset) (Pan et al.). For biosensors, which often generate complex imaging data like fluorescence microscopy or endoscopic images, such algorithms are indispensable for automating analysis, reducing human error, and highlighting critical features like tissue boundaries or abnormal cells. Crucially, this work underscores that next-generation biosensors must evolve beyond mere data acquisition; they should possess the capacity to therapeutically interact with biological systems they monitor.

Clinical translation remains the ultimate goal, and wireless, wearable biosensors are leading this charge. A wireless neonatal sensor exemplifies the critical balance between technical precision and human factors (Senechal et al.). Their prospective study shows strong agreement with wired systems (bias: 0.04 bpm; 97% clinical concordance per Clark Error Grid), while minimizing wire-related complications. Despite Bluetooth disruptions during kangaroo care, nurse and parent satisfaction remained high. This underscores the viability of wireless biosensors in fragile populations, though stability in high-acuity settings requires further testing. This study addresses key challenges faced by wearable biosensors in terms of signal stability and user acceptance during patient care. It exemplifies how functional devices, when tailored to clinical needs, can transform routine monitoring.

The studies highlighted graphene-based modulatory devices, deep learning-enhanced imaging, intelligent segmentation algorithms, and clinical-grade wearables (Jin et al., 2020). It collectively paints a picture of functional devices and biosensors as integrated, patient-centered technologies. From material design that speaks to cellular biology, to imaging that captures life’s finest details, to algorithms that make sense of complexity, to devices that fit seamlessly into care, each advance pushes toward a common goal: technologies that are not only functional but transformative (Welch et al., 2021).

**FIGURE 1:**
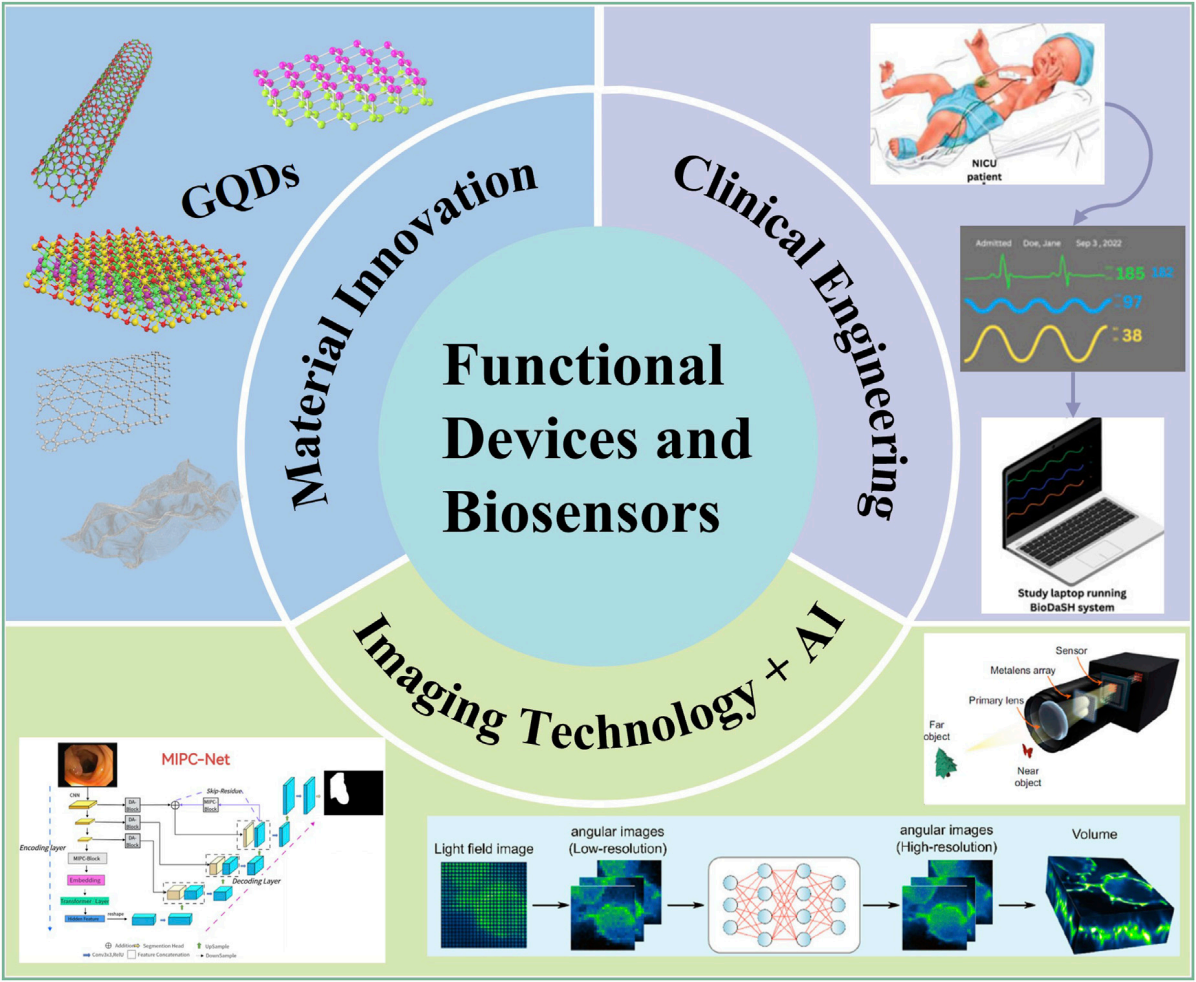
Functional devices and biosensors are evolving along an integrated pipeline from material innovation and intelligent computing to clinical translation, advancing through innovations in graphene quantum dot-based wearables, deep learning-enhanced microscopy, and intelligent segmentation algorithms, to clinically viable wireless neonatal sensors. This convergence is collectively propelling biomedical monitoring and intervention toward precision, real-time operation, and user-centric design.

In summary, the recent progress exemplified by these studies, from graphene-based bioactive devices and AI-enhanced imaging to intelligent data segmentation and clinical-grade wearables, reflecting a multi-disciplinary effort to advance functional devices and biosensors (Figure 1). Future directions should focus on strengthening material-biology interactions, integrating multi-modal sensing like combining electromagnetic and optical readouts, and scaling AI algorithms for diverse biological contexts. By bridging lab innovation and real-world application, these technologies will continue to redefine healthcare, diagnostics and beyond.
